# Prediction of Fontan failure and correlates of Fontan-associated liver disease severity using machine learning and radiomic features from multi-parametric abdominal MRI

**DOI:** 10.1007/s00247-025-06506-w

**Published:** 2026-02-03

**Authors:** Ayush Prasad, Alexander R. Opotowsky, Andrew T. Trout, Lili He, Hailong Li, Jonathan R. Dillman

**Affiliations:** 1 Department of Radiology, Cincinnati Children‘s Hospital Medical Center, 3333 Burnet Avenue, Suite BN6.630, Cincinnati, OH 45229 USA; 2 Heart Institute, Cincinnati Children‘s Hospital Medical Center, Cincinnati, OH 45229 USA

**Keywords:** Fontan operation, MRI, Machine learning, Outcomes, Radiomics, Single ventricle congenital heart disease

## Abstract

**Background:**

Fontan-associated liver disease (FALD) is associated with morbidity and mortality in patients with palliated single ventricle congenital heart disease.

**Objective:**

To develop machine learning models using radiomic features from T1-weighted, T2-weighted, and diffusion-weighted MRI with pertinent clinical variables to predict Fontan failure and correlates of FALD severity in patients who underwent the Fontan operation.

**Materials and methods:**

In this retrospective study of abdominal MRI examinations and clinical record data from 131 Fontan palliation patients (age range 9.1 - 53.3 years old), radiomic features from the liver and spleen were extracted using axial T1-weighted, T2-weighted fat-suppressed, and diffusion-weighted sequences. Patients were categorized by a composite clinical outcome (i.e., Fontan failure) and by correlates of FALD severity, including liver shear stiffness and portal hypertension. Support vector machine (SVM) and multivariable logistic regression models were used to perform two-class classification using radiomic features and/or clinical data. All models were trained and evaluated using five-fold cross-validation (CV).

**Results:**

The best radiomic-only model utilized T2-weighted imaging of both organs with logistic regression to predict the presence of portal hypertension, achieving an AUROC of 0.85±0.01. Clinical-only models showed inferior diagnostic accuracy with the highest AUROC of 0.70±0.08. Combining radiomic and clinical features also did not enhance performance compared to radiomic-only models, with the highest AUROC of 0.77±0.05. Ensemble modeling, which incorporated radiomics from all three MRI sequences, yielded AUROCs ranging from 0.33 to 0.72.

**Conclusion:**

Models incorporating radiomic features from abdominal MRI in Fontan circulation patients demonstrate moderate diagnostic performance for predicting Fontan failure as well as correlates of FALD severity. These models outperformed models containing only clinical electronic health record data and did not improve with ensembled radiomic and clinical data.

**Graphical Abstract:**

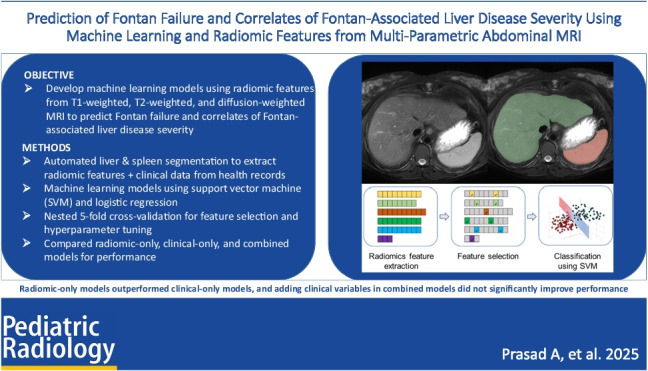

**Supplementary information:**

The online version contains supplementary material available at 10.1007/s00247-025-06506-w.

## Introduction

The Fontan operation is a palliative surgical procedure used in patients with single ventricle forms of congenital heart disease. The procedure diverts the systemic venous blood directly to the pulmonary arteries, bypassing the right side of the heart [1]. Despite its success in improving the life expectancy of these patients, the procedure has been associated with significant long-term complications, and some reports have estimated that up to half of Fontan patients have a major adverse event before adulthood [2].

Fontan-associated liver disease (FALD) is among the procedure’s complications due to chronic passive congestion, as well as other factors, leading to liver injury and fibrosis. Worsening liver fibrosis can lead to portal hypertension, end-stage liver disease, and focal liver lesions including hepatocellular carcinoma. Thus, FALD is an important source of extra-cardiac morbidity and mortality in surgically-repaired single ventricle congenital heart disease [3, 4]. Early detection of FALD and prediction of its clinical course may help prevent adverse outcomes. Liver biopsy is currently considered the reference standard for the diagnosis of FALD, although it is uncommonly performed due to its invasiveness, risk of complications, and need for sedation or anesthesia in children. Further, liver biopsy in those with a Fontan circulation can provide variable results given increased likelihood of sampling-error in the setting of patchy, heterogeneous liver disease. Non-invasive imaging techniques, such as magnetic resonance imaging (MRI), have been increasing in clinical use alongside liver biopsy for diagnosing and monitoring FALD [4, 5].

Machine learning methods used in conjunction with medical images are increasingly described in the literature to improve diagnosis and predict outcomes. Radiomics is a rapidly evolving field that applies the quantitative analysis of medical images to extract latent features, potentially uncovering biologically-meaningful characteristics unappreciated by the human eye [6]. Such features are commonly used as the input for machine learning models. While readily-available MRI-based quantitative markers, such as liver stiffness and liver volume, have shown some ability to predict outcomes in the Fontan circulation [7, 8], we hypothesize that machine learning methods incorporating radiomic features may demonstrate superior performance.

The aim of this study was to use machine learning models and MRI-based radiomic features from the liver and spleen extracted from multi-parametric clinical abdominal MRI examinations as well as pertinent clinical electronic health record data to predict Fontan failure and correlates of FALD severity in children and adults that have undergone Fontan palliation. Predictive models have the potential to identify patients at higher risk of developing impending complications, thereby allowing for early interventions and more personalized management.

## Materials and methods

This retrospective study was HIPAA-compliant and approved by our Institutional Review Board. The requirement for participant informed consent was waived.

### Study sample

**Fig. 1:**
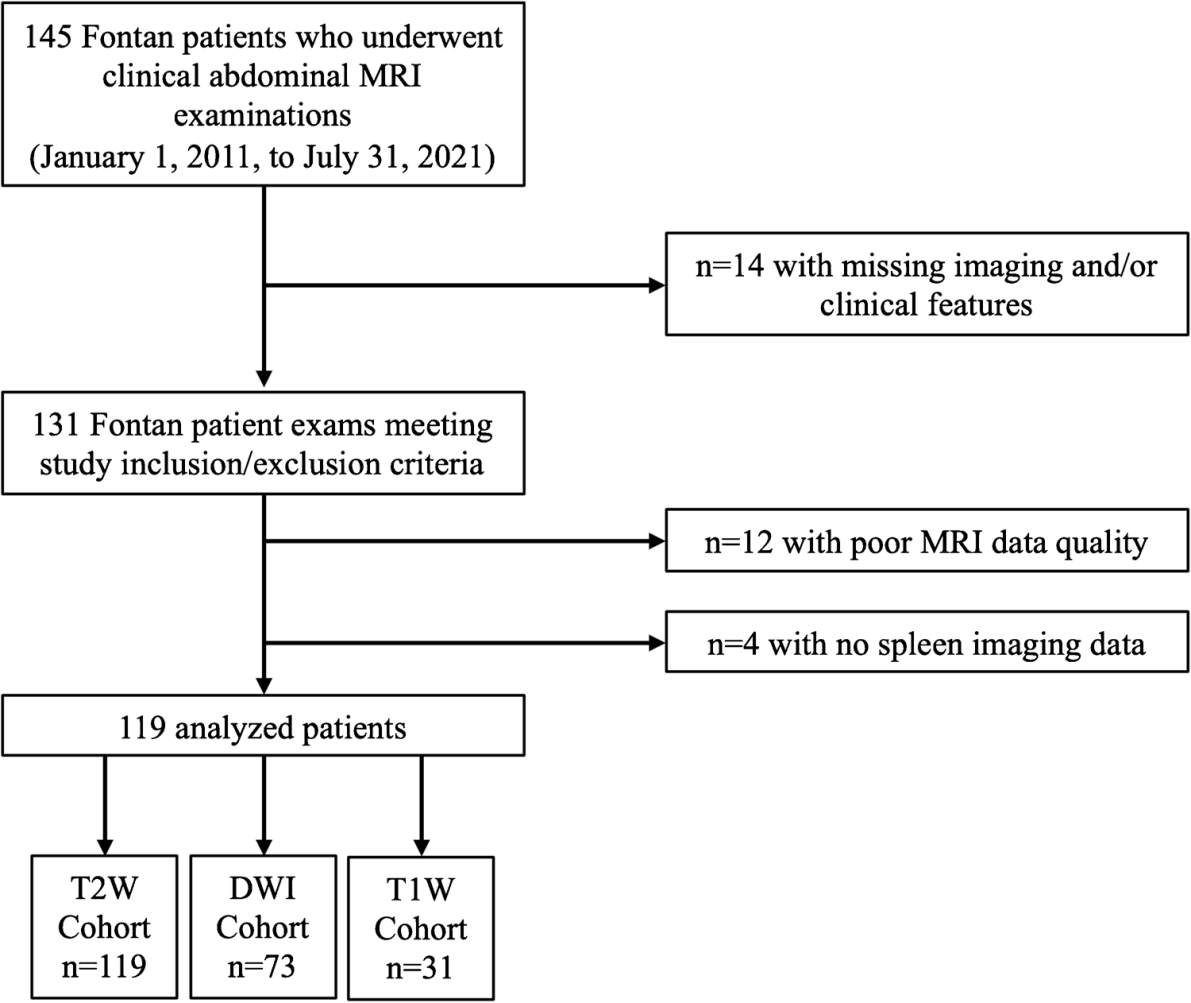
Study participant flow diagram. Poor MRI data quality was defined as exams with severe motion artifacts

Department of Radiology records were searched from January 1, 2011, through July 31, 2021, to identify all clinical abdominal MRI examinations performed in children and adults with single ventricle heart disease who had undergone the Fontan operation (*n*=145). If a patient had undergone multiple MRI examinations following Fontan palliation, the earliest imaging study was used for this investigation, with subsequent examinations excluded. We excluded patients (*n*=14) from this initial search based on missing imaging and/or clinical features as described in the following sections. Moreover, patients with MRI examinations degraded by severe motion artifacts also were excluded from our study (*n*=12). A participant flow diagram is presented in Fig. 1.

### Clinical abdominal MRI examinations

DICOM images from abdominal MRI examinations meeting study inclusion and exclusion criteria were subsequently deidentified and placed on a departmental research server (*n*=131). Of these 131 MRI examinations, 88 (67.2%) were performed on a 1.5-T Ingenia MRI system (Philips Healthcare), 38 (29.0%) were performed on a 1.5-T SIGNA HDxt MRI system (GE HealthCare), 3 (2.3%) were performed on a 1.5-T Optima MR450w MRI system (GE HealthCare), and 2 (1.5%) were performed on a 3.0-T SIGNA Architect MRI system (GE HealthCare). Images analyzed and included in our study included axial 3D Dixon water-weighted (i.e., T1-weighted [T1W]) gradient echo acquired using radial k-space filling and navigator gating, axial 2D T2-weighted (T2W) fast spin-echo (MultiVane XD or PROPELLER) with fat suppression and respiratory triggering, and axial diffusion-weighted (DWI) with respiratory triggering (diffusion *b*-values of 0, 100, and 800), as available. T1W images were available in 33/131 (37.5%) patients, T2W images were identified in 126/131 (96.2%) patients, and DWI sequences were available in 79/131 (60.3%) patients. Pulse sequence details are presented as Supplemental Methods. Not all MRI sequences were available for all patients because abdominal MRI protocols evolved over the study period and varied by scanner manufacturer and clinical indication. As a result, sequence availability reflected real-world clinical variability rather than systematic bias.

### Non-imaging clinical electronic health record variables

For each participant, 27 non-imaging clinical features were retrieved from institutional electronic health records (Epic Hyperspace; Epic Systems Corporation, Verona, WI). These features related to three overarching data domains: demographic and anthropomorphic (e.g., age, sex, body mass index), cardiac medical and surgical history (e.g., specific cardiac anatomy, open fenestration of the Fontan pathway, number of surgical procedures), and pertinent laboratory data (e.g., alanine aminotransferase [ALT], aspartate aminotransferase [AST], bilirubin, albumin). The value closest to the time of the study-related abdominal MRI examination was recorded when multiple values were available. A complete list of the clinical features retrieved for study participants is presented as Supplemental Methods.

### Outcomes, including Fontan failure and measures of FALD severity

Patients were categorized into groups based on three meaningful clinical variables, including (1) development of Fontan circulatory failure within 2 years of this imaging study (yes or no), (2) presence of portal hypertension using the VAST score at the time of this MRI (0–4, calculated by fellowship-trained dedicated pediatric abdominal radiologists at our institution based on the presence of varices, ascites, splenomegaly, and thrombocytopenia, where scores of 2–4 were considered indicative of portal hypertension), and (3) severity of liver shear stiffening (<4.5 kPa vs. ≥4.5 kPa) measured by MR elastography at the time of this MRI. Fontan circulatory failure was defined as a composite outcome that included death, cardiac transplantation, need for ventricular assist device, and/or unanticipated Fontan-related hospitalization occurring within 2 years of the imaging study. A VAST score ≥2 has been associated with an increased odds of experiencing a major adverse event, including death, cardiac transplantation, and hepatocellular cancer [9]. MR elastography was performed using previously described methods [10].

### Radiomic feature extraction

Automated volumetric segmentation of the liver and spleen was performed on axial T1W, T2W, and DWI MR images from each patient, as available, using MRSegmentator—an open-source, robust deep learning model designed for multi-modality segmentation of various anatomical structures [11]. DWI datasets were split into their respective *b*-value imaging volumes, and the MRSegmentator model was applied to the images of the highest *b*-value (*b*=800). The resulting regions of interest (ROIs) for both organs were manually reviewed and corrected for accuracy by a fellowship-trained dedicated pediatric abdominal radiologist (J.R.D., 15 years of post-training experience). Manual edits were limited to small boundary adjustments from the single reviewer in regions affected by organ interface ambiguity, and major re-segmentation was not required. The corresponding voxels (and their raw signal intensities) were obtained from study images.

One-hundred MRI radiomic features were then extracted from the entire liver and spleen imaging volumes, respectively, using PyRadiomics (version 3.1, PyRadiomics Community). Radiomic features included 14 geometric (i.e., shape) features, 18 histogram (first-order) features of signal intensity distribution, 14 texture features from the gray-level dependence matrix, 22 texture features from the gray-level cooccurrence matrix, 16 texture features from the gray-level run-length matrix, and 16 texture features from the gray-level size-zone matrix. Before feature extraction, PyRadiomics also was used to normalize voxel intensities to a range of –300 to 300 and then resample voxels to a resolution of 1 mm×1 mm×1 mm. These resampling dimensions were chosen after confirmation of minimal effect of resampling by testing smaller values on feature robustness and model performance.

### Machine learning models

Conventional machine learning models were used to predict the three clinical variables described above. The support vector machine (SVM) algorithm, a supervised machine learning algorithm that maximizes the distance of a separating hyperplane between two groups, was used to construct classification models [12]. These models were built using scikit-learn version 1.5.2 (https://www.scikit-learn.org), a Python library for machine learning. Both linear and nonlinear SVM models were tested, with nonlinear SVM models developed using a radial-basis function (RBF) kernel. SVM coefficients were also retrieved to determine the discriminative power of individual radiomic and clinical features. Conventional statistical models, including multivariable logistic regression, were also employed, with their results compared to our SVM models. To assess the complementary value of radiomic features derived from T1W, T2W, and DWI, we also evaluated ensemble models by implementing a simple “voting” metaclassifier. We averaged probability estimates from models trained separately on each sequence for each clinical variable from the cohort of patients with all three MRI sequences to produce a consensus score. The ensemble models are further described in Supplemental Methods.

All machine learning model inputs included abdominal MRI radiomic and clinical features. Classification models were built using four general approaches: (i) radiomic features from the liver, (ii) radiomic features from the spleen, (iii) radiomic features from both the liver and spleen, and (iv) clinical features alone. In addition, we built models using combinations of radiomic and clinical features.

### Model training, optimization, and evaluation

**Fig. 2:**
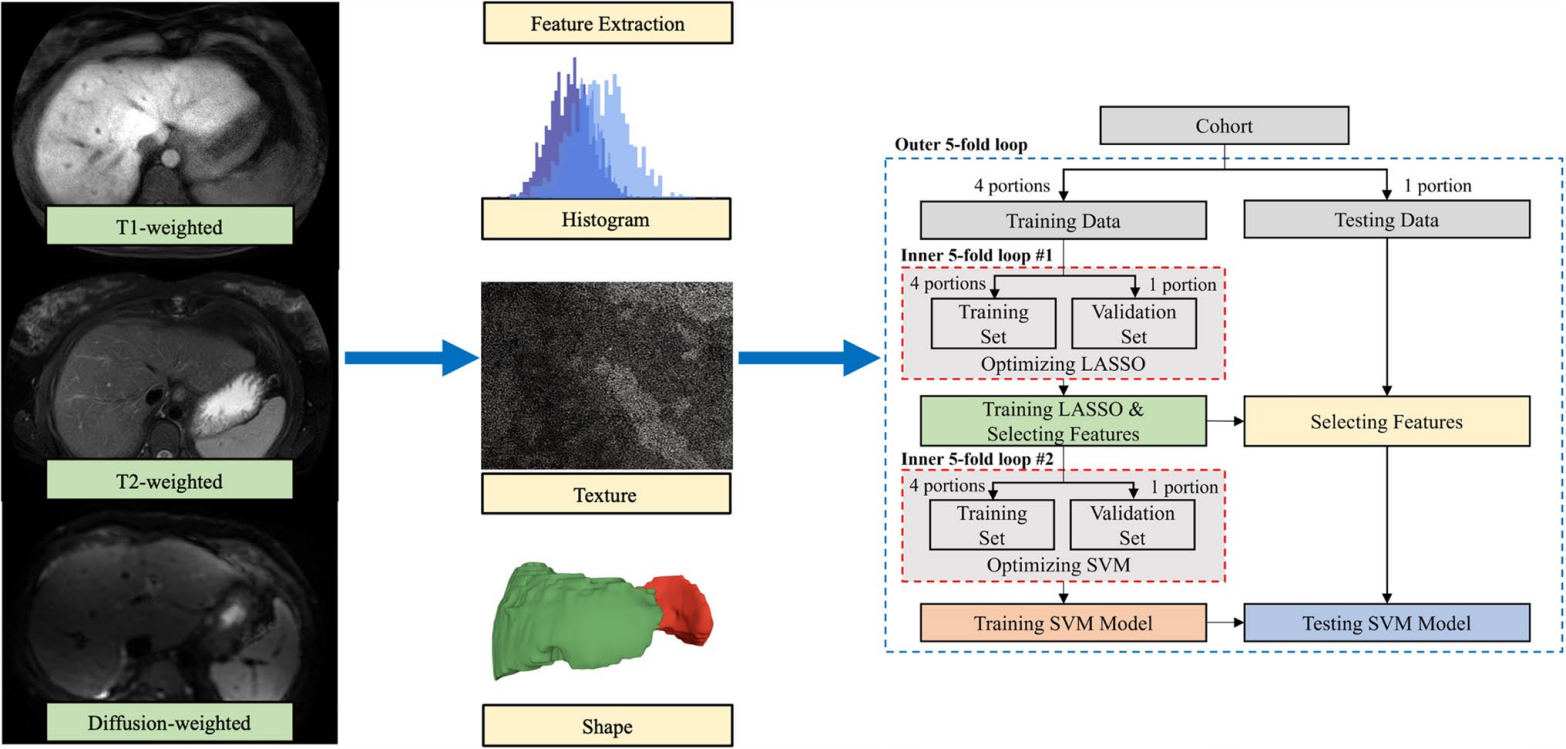
Flow diagram of machine learning methods. The nested 5-fold CV includes an outer loop and 2 inner loops. The outer loop splits the dataset into training (4 portions) and testing (1 portion) data across 5 iterations. Two inner loops optimize LASSO for feature selection, with training data further divided into training and validation sets. Final model performance is reported based on the outer loop results after 5 iterations

Feature selection was performed using least absolute shrinkage and selection operator (LASSO) regression, which reduces the number of features and improves the prediction accuracy of machine learning models [13]. A nested 5-fold cross-validation (CV) strategy was used to train and evaluate the machine learning models. Specifically, within each outer-loop iteration, we applied two inner loops to the development data. The first inner loop was used to optimize feature selection using LASSO regression. The development data was further divided into training (four portions) and validation (one portion) sets in a 5-fold manner. LASSO was applied to the training set to identify the most discriminative radiomic and/or clinical features, and performance was evaluated on the corresponding validation set. In the second inner loop, we optimized the hyperparameters of the support vector machine (SVM) model, particularly the penalty coefficient (*C*). Again, the development data was split into training and validation sets, with models trained on the training set and evaluated on the unseen validation set. Final model performance was reported based on the outer loop, using predictions on the held-out testing set from each of the five iterations. A flow diagram summarizing our machine learning methods is outlined in Fig. 2.

Model results were evaluated using area under the receiver operating characteristic curve (AUROC). To assess the robustness and generalizability of each model, we repeated the nested 5-fold CV process 50 times using different random splits. For each model architecture, we calculated the mean and standard deviation of all performance metrics across these repetitions.

### Statistical analyses

Continuous data were summarized using means, standard deviations, and ranges, while categorical data were summarized using counts and percentages. Differences between patient groups were compared using the Wilcoxon rank sum test for continuous variables, while the Fisher exact test was used to compare categorical variables. A *P*-value <0.05 was considered significant for all inference testing. Statistical analyses were conducted using Python software (version 3.11.9).

## Results

### Study sample and clinical variables

Our final study sample included 119 patients (mean age=20.4±7.6 years; 54 [45.4%] female); 68/119 (57.1%) of whom were 18 years old or older at the time of their abdominal MRI examination (age range, 9.1–53.3 years). Baseline characteristics of the participants are presented in Table 1 as well as in Supplemental Table 1.

**Table 1:** Baseline participant characteristics, including sub-cohorts based on MRI pulse sequence availability

	T2-weighted (*n*=119)	Diffusion-weighted (*n*=73)	T1-weighted (*n*=31)	*P*
Sex=female (%)	54 (45.4)	35 (47.9)	11 (35.5)	0.498
Age at time of MRI (mean [SD])	20.43 [7.57]	20.85 [7.35]	20.12 [6.33]	0.876
BSA at time of MRI (mean [SD])	1.70 [0.33]	1.71 [0.34]	1.70 [0.33]	0.994
Underlying cardiac anatomy (%)				0.916
TA	34 (28.6)	21 (28.8)	6 (19.4)	
DILV	25 (21.0)	14 (19.2)	5 (16.1)	
HLHS	33 (27.7)	23 (31.5)	11 (35.5)	
PA-IVS	3 (2.5)	2 (2.7)	2 (6.5)	
Other	24 (20.2)	13 (17.8)	7 (22.6)	
Fontan procedure type (%)				0.793
Atriopulmonary	12 (10.2)	9 (12.5)	1 (3.3)	
Lateral tunnel	46 (39.0)	27 (37.5)	14 (46.7)	
Extracardiac	59 (50.0)	36 (50.0)	15 (50.0)	
Fontan open fenestration (%)	91 (77.8)	55 (76.4)	26 (83.9)	0.692
Hypoplastic ventricle (%)				0.66
Left	65 (54.6)	37 (50.7)	15 (48.4)	
Right	52 (43.7)	34 (46.6)	14 (45.2)	
Codominant	2 (1.7)	2 (2.7)	2 (6.5)	
Number of cardiac surgeries (mean [SD])	2.99 [0.70]	2.99 [0.64]	2.97 [0.85]	0.986
Current smoking (%)	10 (8.4)	5 (6.8)	2 (6.5)	0.893

*BSA* body surface area, *TA* truncus arteriosus, *DILV* double inlet left ventricle, *HLHS* hypoplastic left heart syndrome, *PA-IVS* pulmonary atresia with intact ventricular septum

Regarding our clinical variables of interest, 30/119 (25.2%) patients were diagnosed with Fontan circulatory failure, 26/119 (21.8%) patients had moderate-severe portal hypertension with a VAST score of 2–4, and 53/119 (44.5%) patients demonstrated an MR elastography liver shear stiffness ≥4.5 kPa (Table 2).

**Table 2:** Clinical variables of interest, including Fontan circulatory failure, based on MRI pulse sequence availability

	T2-weighted (*n*=119)	Diffusion-weighted (*n*=73)	T1-weighted (*n*=31)	*P*-value
Fontan circulatory failure (*n*, %)	30 (25.2)	20 (27.4)	6 (19.4)	0.69
Death (*n*, %)	7 (5.9)	5 (6.8)	0 (0.0)	0.35
Need for liver transplant (*n*, %)	6 (5.0)	5 (6.8)	2 (6.5)	0.86
VAD placement (*n*, %)	2 (1.7)	2 (2.7)	1 (3.2)	0.82
Unanticipated hospitalization (*n*, %)	29 (24.4)	19 (26.0)	6 (19.4)	0.77
VAST score ≥2 (*n*, %)	26 (21.8)	16 (21.9)	7 (22.6)	0.99
Liver shear stiffness ≥4.5 kPa (*n*, %)	53 (44.5)	28 (38.4)	13 (41.9)	0.72

*VAD* ventricular assist device, *VAST* varices, ascites, splenomegaly, thrombocytopenia

### Model performance

In total, 108 unique models were explored using liver and/or spleen radiomic features derived from T1W, T2W, and/or DWI MR images with and without clinical features and three model architectures (linear SVM, nonlinear SVM, and logistic regression), and across the three clinical variables of interest. For the nested 5-fold cross-validation design, in each outer fold, approximately 80% of the cohort (e.g., about 95/119 patients for the T2W cohort) served as the development set and 20% (e.g., about 24/119 patients for the T2W cohort) served as the held-out test set. Within the development set, two additional 5-fold inner loops divided the data (e.g., about 76/95 patients for training and 19/95 for validation for the T2W cohort) during feature selection and hyperparameter tuning. This process ensured that all patients contributed to both training and testing across the cross-validation cycles.

### Radiomic-only model performance

Radiomic-only models demonstrated varying performance depending on both the imaging sequence and the organ used for feature extraction for the three clinical variables. The diagnostic performances of all radiomic-only models are presented in Table 3.

**Table 3:**
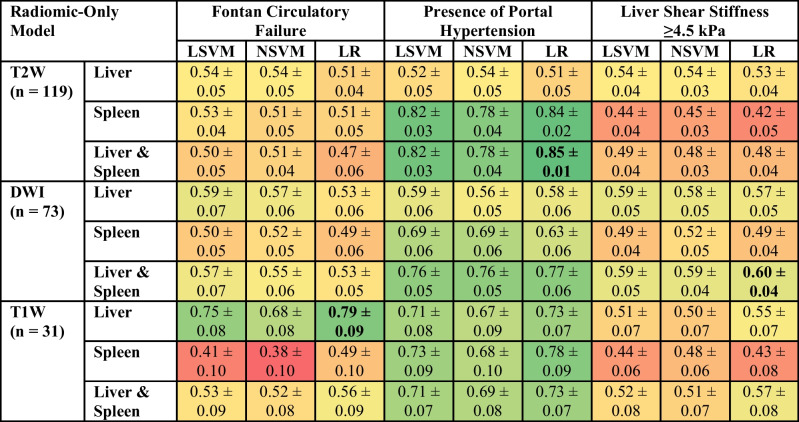
Diagnostic performance of radiomic-only models presented as area under the receiver operating characteristic curve (AUROC). The models with the best performance for each variable of interest are in bold. The table is color-coded as a heat map with values approaching 1 as greener shades in color and those nearing 0 as redder shades in color

*SVM* support vector machine, *LSVM* linear SVM, *NSVM* nonlinear SVM, *LR* logistic regression, *T2W* T2-weighted imaging, *T1W* T1-weighted imaging, *DWI* diffusion-weighted imaging

Among single-organ models, liver-based radiomics achieved comparable or higher AUROC values compared to spleen-based models across almost all imaging sequences and clinical variables. The best-performing single-organ model for predicting the Fontan circulatory failure classification task used logistic regression modeling of T1W imaging data from the liver, demonstrating an AUROC of 0.79±0.09. However, T2W imaging data of the spleen performed better across model types compared to the corresponding liver imaging data (AUROC 0.78–0.84 vs. 0.51–0.54) when predicting the presence of portal hypertension. More specifically, T2W imaging of the spleen using logistic regression was the best-performing single-organ model for this clinical variable (AUROC 0.84±0.02). For predicting higher liver stiffness (≥4.5 kPa), single-organ models demonstrated limited performance across imaging  sequences (AUROC 0.42–0.59).

The liver-spleen radiomic models generally matched or outperformed the single-organ models, with the highest performance observed using T2W imaging and logistic regression modeling to predict the presence of portal hypertension, achieving an AUROC of 0.85±0.01. The classification task for predicting Fontan circulatory failure using liver-spleen models demonstrated limited performance across imaging sequences (AUROC 0.47–0.57). Lastly, the best-performing model for predicting a higher liver stiffness (≥4.5 kPa) used DWI imaging data from both the liver and spleen with logistic regression modeling, demonstrating an AUROC of 0.60±0.04.

### Clinical-only model performance

Clinical-only models generally yielded lower AUROC values when compared against radiomic-only and/or combination models for the three clinical variables. The diagnostic performances of all clinical models are presented in Table 4. The best-performing clinical-only model for predicting Fontan circulatory failure was in the DWI imaging cohort, demonstrating an AUROC of 0.60±0.05 with logistic regression modeling. The best-performing clinical-only model for predicting the presence of portal hypertension demonstrated an AUROC of 0.70±0.08 in the T1W imaging cohort and logistic regression modeling. This imaging cohort and model technique also provided the best-performing clinical-only model for predicting a higher liver stiffness (≥4.5 kPa), demonstrating an AUROC of 0.63±0.07.

**Table 4:**
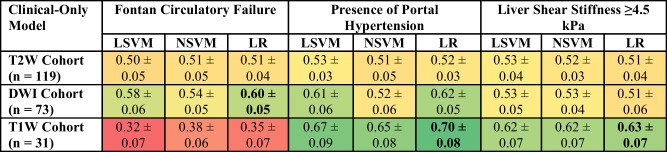
Diagnostic performance of clinical-only models presented as area under the receiver operating characteristic curve (AUROC). The models with the best performance for each variable of interest are in bold. The table is color-coded as a heat map with values approaching 1 as greener shades in color and those nearing 0 as redder shades in color

*SVM* support vector machine, *LSVM* linear SVM, *NSVM* nonlinear SVM, *LR* logistic regression, *T2W* T2-weighted imaging, *T1W* T1-weighted imaging, *DWI* diffusion-weighted imaging

### Combination model performance

Combination models with both radiomic *and* clinical features failed to consistently improve model performance across the three clinical variables of interest when compared to radiomic-only models. The diagnostic performances of all combination models are presented in Table 5. As seen with the radiomic-only single-organ models, combination models with liver imaging data achieved comparable or higher AUROC values compared to models with spleen imaging data across almost all imaging sequences and clinical variables. However, T2W imaging data of the spleen with clinical data performed better across model types compared to the corresponding combination model with liver imaging data (AUROC 0.72–0.76 vs. 0.52–0.55) when predicting the presence of portal hypertension.

**Table 5:**
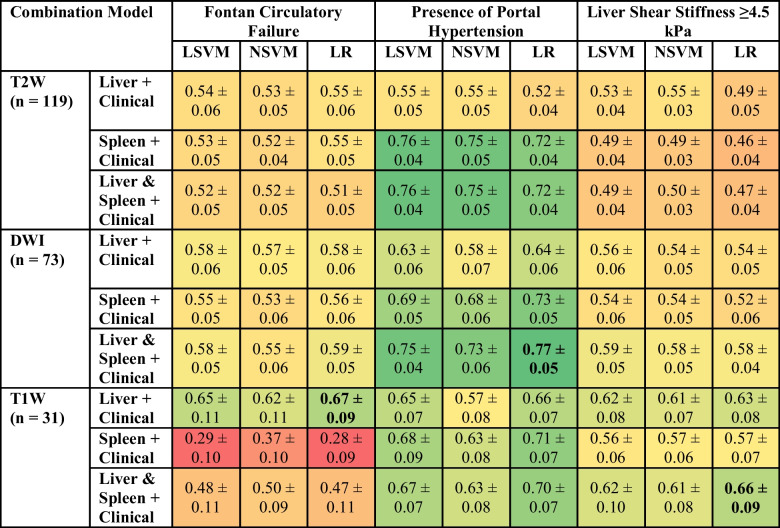
Diagnostic performance of the radiomic and clinical combination models presented as area under the receiver operating characteristic curve (AUROC). The models with the best performance for each variable of interest are in bold. The table is color-coded as a heat map with values approaching 1 as greener shades in color and those nearing 0 as redder shades in color

*SVM* support vector machine, *LSVM* linear SVM, *NSVM* nonlinear SVM, *LR* logistic regression, *T2W* T2-weighted imaging, *T1W* T1-weighted imaging, *DWI* diffusion-weighted imaging

The combination models with liver-spleen radiomics generally matched or outperformed the single-organ combination models, with the highest performance observed using DWI imaging and logistic regression modeling to predict the presence of portal hypertension, achieving an AUROC of 0.77±0.05. The best-performing combination model for the Fontan circulatory failure classification task used logistic regression modeling with T1W imaging data from the liver and clinical data, demonstrating an AUROC of 0.67±0.09. T1W imaging of both the liver and spleen with clinical data was again shown to perform worse in predicting Fontan circulatory failure when compared to the corresponding liver-only combination model (AUROC 0.47–0.50 vs. 0.62–0.67). The best-performing combination model for predicting a higher liver stiffness (≥4.5 kPa) used T1W imaging data from the liver and spleen with logistic regression modeling, demonstrating an AUROC of 0.66±0.09.

The top ten predictive radiomic and clinical features from the best-performing combination models using both liver and spleen imaging data are presented in Table 6.

**Table 6:** Top ten most predictive radiomic and clinical features from the best-performing combination models using both liver and spleen imaging data for each clinical variable of interest

	Fontan circulatory failure (DWI+LR) AUROC=0.59±0.05	Presence of portal hypertension (DWI+LR) AUROC=0.77±0.05	Liver shear stiffness ≥4.5 kPa (T1W+LR) AUROC=0.66±0.09
#1	Fontan procedure type (atriopulmonary, lateral tunnel, extracardiac)	Least axis length – liver shape	Sex
#2	Creatinine	Sphericity – spleen shape	Fontan procedure type
#3	MELD-XI	APRI	APRI
#4	Maximum probability – liver texture	Sphericity – liver shape	10th percentile – liver voxel intensities
#5	ALT	ALT	Minimum – spleen voxel intensities
#6	Median – liver voxel intensities	Flatness – liver shape	Mean – liver voxel intensities
#7	Triglycerides	Weight at time of MRI	Median – liver voxel intensities
#8	INR	AFP	Minimum – liver voxel intensities
#9	Underlying cardiac anatomy	Large dependence high gray level emphasis – spleen texture	Root mean squared – liver voxel intensities
#10	Gray-level nonuniformity – liver texture	Total bilirubin	Gray-level nonuniformity normalized – liver texture

*LR* logistic regression, *T2W* T2-weighted imaging, *T1W* T1-weighted imaging, *INR* international normalized ratio, *ALT* alanine aminotransferase, *AST* aspartate aminotransferase, *MELD-XI* model for end-stage liver disease excluding INR, *APRI* AST to platelet ratio index, *AFP* alpha-fetoprotein

### Ensemble model performance

Ensemble models using radiomics from all three MRI sequences with clinical models demonstrated lower AUROC values when compared against radiomic-only and/or combination models for the three clinical variables. The diagnostic performances of ensemble models are presented in Table 7.

**Table 7:**
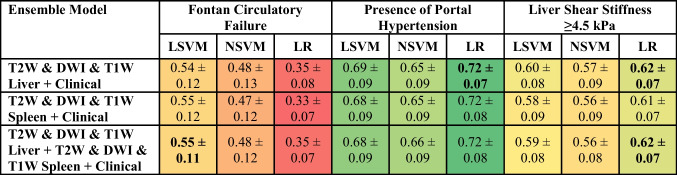
Diagnostic performance of radiomic-clinical ensemble models presented as area under the receiver operating characteristic curve (AUROC). The models with the best performance for each variable of interest are in bold. The table is color-coded as a heat map with values approaching 1 as greener shades in color and those nearing 0 as redder shades in color

*SVM* support vector machine, *LSVM* linear SVM, *NSVM* nonlinear SVM, *LR* logistic regression, *T2W* T2-weighted imaging, *T1W* T1-weighted imaging, *DWI* diffusion-weighted imaging

## Discussion

To our knowledge, this is the first investigation involving the application of machine learning models to clinical abdominal MR images and electronic health record clinical data to predict Fontan failure and measures of FALD severity in pediatric and adult patients post-Fontan. Radiomic-only models generally outperformed clinical-only and combination radiomic-clinical models for the prediction of the three clinical variables. For predicting Fontan circulatory failure, the liver-only radiomic model using T1W imaging and logistic regression achieved the highest performance (AUROC 0.79±0.09). The best-performing model for predicting the presence of portal hypertension was the liver-spleen radiomic model using T2W imaging with logistic regression (AUROC 0.85±0.01). The best model for predicting higher liver stiffness (≥4.5 kPa) was the combination model with both liver-spleen imaging data and clinical data using T1W imaging with logistic regression (AUROC 0.66±0.09). Ensemble models, which combined radiomics from all MRI sequences with clinical data, did not improve diagnostic performance compared to the single-sequence or combination models.

While our models show that liver and spleen radiomic features are associated with the development of Fontan circulatory failure, portal hypertension, and liver stiffness, the overall moderate performance of these models suggests that the complexity of the Fontan circulation may not be fully captured by our current set of radiomic features and/or clinical data. For example, the difficulties in predicting different Fontan-related outcomes and measures of severity may be attributed to the complexity of factors influencing each clinical variable, including the diverse nature of complications such as death, cardiac transplantation, ventricular assist device requirement, and hospitalization that defined our composite outcome for Fontan circulatory failure. Moreover, single-organ, radiomic-only models demonstrated limited performance across imaging sequences for predicting higher liver stiffness ≥4.5 kPa (AUROC 0.42–0.59), highlighting the importance of magnetic resonance elastography (MRE) in this population.

The three variables (i.e., Fontan failure, severity of portal hypertension, and severity of liver stiffening) evaluated in this work represent different aspects of the spectrum of FALD. Because these processes do not always progress in parallel, patients may exhibit abnormalities in one domain without corresponding changes in another. These distinctions likely explain, at least in part, the variation in model performance across clinical variables, with imaging-based radiomic features more strongly linked to the presence of portal hypertension than to the broader, multifactorial construct of Fontan circulatory failure. Moreover, several of the top radiomic features identified in our models have plausible biological relevance to FALD. Shape metrics such as sphericity and flatness may reflect organ enlargement or distortion from chronic venous congestion and tissue fibrosis, while texture features (e.g., gray-level nonuniformity, dependence, and size-zone measures) capture increasing parenchymal heterogeneity associated with congestion, fibrosis, vascular remodeling, and altered perfusion. Intensity-based features may similarly reflect changes in parenchymal composition, such as congestion and fibrosis. Ultimately, these relationships provide a pathophysiologic basis for why these features were predictive, particularly for the presence of portal hypertension, and highlight how radiomics can quantify subtle tissue remodeling that is not easily appreciated by the human eye on routine imaging.

Clinical-only models achieved AUROCs ranging from 0.32 to 0.70, suggesting their limited and unpredictable standalone discriminatory capacity for predicting our clinical variables of interest. The use of both radiomic and clinical features failed to significantly improve prediction of Fontan failure and correlates of FALD severity when compared to radiomic features alone.

Moreover, model performance did not improve when radiomic data across all three MRI sequences were ensembled together with clinical data, with ensemble models demonstrating AUROCs ranging from 0.33 to 0.72. The lower performance of the ensemble models was likely driven by the inclusion of lower-performing sequence-specific models that diluted the stronger signal from higher-performing models. Because T1W and DWI models showed limited discrimination, averaging their predictions with the higher-performing T2W models likely reduced overall accuracy. The clinical model additionally showed comparatively modest discriminative performance. Next, the ensemble analyses were restricted to the smaller subset of patients with all three sequences (*n*=31), increasing variance and reducing stability. Lastly, radiomic features across sequences may capture overlapping or partially conflicting information, limiting the benefit of combining them. If the ensemble weighting scheme is not sufficiently adaptive, dominant but less-informative features can overshadow more predictive features. These considerations suggest that more information does not necessarily translate to improved performance unless each component of the model contributes robust, non-redundant predictive value.

Our machine learning models incorporating clinical and/or radiomic features have demonstrated moderate accuracy and AUC values, performing at around the same level as previous work in predicting liver stiffness in pediatric and young adult patients with known or suspected liver disease with similar methods [14]. Alsaied et al. previously showed MRE-derived liver stiffness to have moderate predictive power for Fontan failure and portal hypertension, with a reported sensitivity and specificity around 77% [15]. Liver volume indexed to body surface area has been linked to adverse cardiovascular outcomes in Fontan circulation patients, but its predictive accuracy is less robust compared to the above methods [16]. Lastly, various identified hemodynamic and clinical parameters have been described as predictors of Fontan failure, but these methods generally lack the predictive precision and integration capabilities of machine learning approaches [17]. Our findings suggest that the addition of radiomic features to models may provide a complementary, non-invasive tool to enhance risk stratification in FALD patients by capturing subtle textural and morphologic changes associated with congestion, fibrosis, and portal hypertension.

We believe machine learning models, particularly when developed from a larger study sample, will provide a more comprehensive and accurate prediction of Fontan circulation failure by integrating diverse data sources and leveraging advanced algorithms. While the AUROC of 0.85±0.01 for predicting the presence of portal hypertension is encouraging and approaches a clinically useful level, the more modest performance of other models in this work is insufficient for direct clinical action. These results should therefore be viewed as early exploratory steps, highlighting potential imaging-derived biomarkers rather than actionable clinical tools. In practice, a risk-prediction model for clinical use would require consistently strong discrimination (AUROC>0.80) with external validation before meaningfully influencing surveillance and/or management decisions in Fontan patients.

The potential of machine learning models to predict future or impending complications in Fontan patients could have clinical implications. Such models could help identify early warning signs of clinical deterioration, enabling proactive interventions and personalized care strategies tailored to the unique physiology and complications of these patients. Moreover, these models offer the potential to extract additional information from routine abdominal MRI scans that may currently go unnoticed by the reading radiologist. By uncovering subtle imaging biomarkers or patterns, this approach could enhance our understanding of the Fontan circulation and its sequelae. As these models evolve, they may ultimately serve as a valuable tool in guiding clinical decision-making, optimizing follow-up care, and improving overall patient outcomes for this population.

Our study has limitations. First, all models were trained and validated on MR images and clinical data from a single institution, and the lack of an available independent external test dataset is a noteworthy limitation with relevance. Future efforts are needed to further validate these models using external data as opposed to the cross-validation methodology employed for our study. Second, we incorporated handcrafted radiomic features into our models as opposed to employing deep learning feature extraction. Deep learning-based features may further enhance the diagnostic performance of such models by capturing many more magnitudes of latent data to identify even subtle patterns and nuances in medical images, particularly with increasing sample sizes. For example, we have previously shown similar performance of the liver stiffness classification task using deep learning compared to models using radiomic features in a different only slightly larger pediatric and young adult cohort [18]. Third, our study used radiomic features from only three clinical MRI sequences (i.e., image contrasts), some of which were missing for certain patients. Multi-parametric imaging data is not always available for every patient, and it is conceivable that radiomic features from other sequences could have superior diagnostic performance to those investigated, although this remains to be shown. Furthermore, it is possible that a larger study sample with less missing imaging data could also improve model performance and improve model robustness. Finally, the integration of more diverse clinical parameters, such as those extracted from the electronic health record with natural language processing models, with longitudinal MRI data may also contribute to improved performance.

## Conclusion

Machine learning models incorporating radiomic features from routine abdominal MRI demonstrate moderate diagnostic performance in predicting Fontan failure and correlates of FALD severity in pediatric and adult patients with Fontan circulation. The addition of selected, discrete electronic health record clinical data to our models did not significantly improve performance. This study helps lay the foundation for efforts to leverage machine learning techniques applied to non-invasive MR images in patients with a Fontan circulation to enhance diagnosis and predict meaningful outcomes, ultimately improving clinical outcomes in this population.

## Supplementary information

Below is the link to the electronic supplementary material.

ESM 1 (DOCX 28.4 KB)

## Data Availability

The data that support the findings of this study are available from the corresponding author, J.R.D., upon reasonable request.

## References

[1] Mazza GA, Gribaudo E, Agnoletti G (2021) The pathophysiology and complications of Fontan circulation. Acta Biomed 92:e2021260. https://doi.org/10.23750/abm.v92i5.10893 34738582 10.23750/abm.v92i5.10893 PMC8689331

[2] Plappert L, Edwards S, Senatore A, De Martini A (2022) The epidemiology of persons living with Fontan in 2020 and Projections for 2030: development of an epidemiology model providing multinational estimates. Adv Ther 39(2):1004–1015. 10.1007/s12325-021-02002-3 10.1007/s12325-021-02002-3 PMC8866255 34936056

[3] Gordon-Walker TT, Bove K, Veldtman G (2019) Fontan-associated liver disease: a review. J Cardiol 74:223–232 30928109 10.1016/j.jjcc.2019.02.016

[4] Dillman JR, Trout AT, Alsaied T, Gupta A, Lubert AM (2020) Imaging of Fontan-associated liver disease. Pediatr Radiol 50:1528–1541. https://doi.org/10.1007/s00247-020-04776-0 32809067 10.1007/s00247-020-04776-0

[5] Emamaullee J, Zaidi AN, Schiano T, Kahn J, Valentino PL, Hofer RE, Taner T, Wald JW, Olthoff KM, Bucuvalas J, Fischer R (2020) Fontan-associated liver disease: screening, management, and transplant considerations. Circulation 142(6):591–604. 10.1161/CIRCULATIONAHA.120.045597 10.1161/CIRCULATIONAHA.120.045597 PMC7422927 32776846

[6] Lambin P, Leijenaar RTH, Deist TM, Peerlings J, de Jong EEC, van Timmeren J, Sanduleanu S, Larue RTHM, Even AJG, Jochems A, van Wijk Y, Woodruff H, van Soest J, Lustberg T, Roelofs E, van Elmpt W, Dekker A, Mottaghy FM, Wildberger JE, Walsh S (2017) Radiomics: the bridge between medical imaging and personalized medicine. Nat Rev Clin Oncol 14:749–762 28975929 10.1038/nrclinonc.2017.141

[7] Lubert AM, Opotowsky AR, Palermo JJ, Alsaied T, Szugye C, Anwar N, Tiao GM, Lorts A, Dillman JR, Trout AT (2021) Relation of liver volume to adverse cardiovascular events in adolescents and adults with Fontan circulation. Am J Cardiol S0002-9149:01107-3 10.1016/j.amjcard.2021.10.045 34893302

[8] Brayer SW, Zafar F, Lubert AM, Trout AT, Palermo JJ, Opotowsky AR, Anwar N, Dillman JR, Alsaied T (2021) Relation of magnetic resonance elastography to Fontan circulatory failure in a cohort of pediatric and adult patients. Pediatr Cardiol 42:1871–1878 34448042 10.1007/s00246-021-02707-w

[9] Elder RW, McCabe NM, Hebson C, Veledar E, Romero R, Ford RM, Mahle WT, Kogon BE, Sahu A, Jokhadar M, McConnell ME, Book WM (2013) Features of portal hypertension are associated with major adverse events in Fontan patients: the VAST study. Int J Cardiol 168(4):3764–9. 10.1016/j.ijcard.2013.06.008 10.1016/j.ijcard.2013.06.008 PMC3805740 23849105

[10] Serai SD, Towbin AJ, Podberesky DJ (2012) Pediatric liver MR elastography. Dig Dis Sci 57:2713–2719 22569825 10.1007/s10620-012-2196-2

[11] Häntze H, Xu L, Mertens CJ, Dorfner FJ, Donle L, Busch F, Kader A, Ziegelmayer S, Bayerl N, Navab N, Rueckert D, Schnabel J, Aerts HJWL, Truhn D, Bamberg F, Weiss J, Schlett CL, Ringhof S, Niendorf T, Pischon T, Kauczor HU, Nonnenmacher T, Kröncke T, Völzke H, Schulz-Menger J, Maier-Hein K, Hering A, Prokop M, van Ginneken B, Makowski MR, Adams LC, Bressem KK (2025) Segmenting whole-body MRI and CT for multiorgan anatomic structure delineation. Radiol Artif Intell 7(6):e240777. 10.1148/ryai.240777 40767616 10.1148/ryai.240777

[12] Cortes C, Vapnik V (1995) Support-vector networks. Mach Learn 20:273–297

[13] Tibshirani R (1996) Regression shrinkage and selection via the lasso. J R Stat Soc Ser B Stat Methodol 58:267–288

[14] He L, Li H, Dudley JA, Maloney TC, Brady SL, Somasundaram E, Trout AT, Dillman JR (2019) Machine learning prediction of liver stiffness using clinical and T2-weighted MRI radiomic data. AJR Am J Roentgenol 213:592–601 31120779 10.2214/AJR.19.21082

[15] Alsaied T, Possner M, Lubert AM, Trout AT, Szugye C, Palermo JJ, Lorts A, Goldstein BH, Veldtman GR, Anwar N, Dillman JR (2019) Relation of magnetic resonance elastography to Fontan failure and portal hypertension. Am J Cardiol 124:1454–1459. 10.1016/j.amjcard.2019.07.052 31474329 10.1016/j.amjcard.2019.07.052

[16] Lubert AM, Opotowsky AR, Palermo JJ, Alsaied T, Szugye C, Anwar N, Tiao GM, Lorts A, Dillman JR, Trout AT (2022) Relation of liver volume to adverse cardiovascular events in adolescents and adults with Fontan circulation. Am J Cardiol 165:88–94 34893302 10.1016/j.amjcard.2021.10.045

[17] Inai K, Inuzuka R, Ono H, Nii M, Ohtsuki S, Kurita Y, Takeda A, Hirono K, Takei K, Yasukouchi S, Yoshikawa T, Furutani Y, Shimada E, Shinohara T, Shinozaki T, Matsuyama Y, Senzaki H, Nakanishi T (2022) Predictors of long-term mortality among perioperative survivors of Fontan operation. Eur Heart J 43:2373–2384. 10.1093/eurheartj/ehab826 34888643 10.1093/eurheartj/ehab826

[18] Li H, He L, Dudley JA, Maloney TC, Somasundaram E, Brady SL, Parikh NA, Dillman JR. DeepLiverNet: a deep transfer learning model for classifying liver stiffness using clinical and T2-weighted magnetic resonance imaging data in children and young adults. Pediatr Radiol. 2021 Mar;51(3):392–402. 10.1007/s00247-020-04854-3 10.1007/s00247-020-04854-3 PMC8675279 33048183

