# Supplementary material for: Prediction of Fontan failure and correlates of Fontan-associated liver disease severity using machine learning and radiomic features from multi-parametric abdominal MRI
Source: Pediatr Radiol. 2026 Feb 3;56(4):831–42. doi: 10.1007/s00247-025-06506-w (PMC13035638; doi:10.1007/s00247-025-06506-w)
Supplement: Supplementary file 1 — (DOCX 28.4 KB) [file 247_2025_6506_MOESM1_ESM.docx]

**SUPPLEMENTAL METHODS**

**Clinical Features**

Demographics features collected were age, sex, height, weight, and body surface area (BSA) at the time of the abdominal MRI. Features pertaining to medical history were history of smoking, underlying cardiac anatomy type (truncus arteriosus [TA], double inlet left ventricle [DILV], hypoplastic left heart syndrome [HLHS], pulmonary atresia with intact ventricular septum [PA-IVS], or other type), Fontan procedure type (atriopulmonary, lateral tunnel, extracardiac, or other type), Fontan conversion (yes or no), Fontan open fenestration (yes or no), hypoplastic ventricle (right, left, or codominant), and prior number of cardiac surgeries. Lastly, laboratory values included in our feature set were creatinine, albumin, alanine aminotransferase (ALT), aspartate aminotransferase (AST), total bilirubin, alkaline phosphatase (ALP), gamma-glutamyl transferase (GGT), international normalized ratio (INR), alpha-fetoprotein (AFP), model for end-stage liver disease excluding INR (MELD-XI, calculated) score, AST to platelet ratio index (APRI, calculated), total cholesterol, low-density lipoprotein (LDL), high-density lipoprotein (HDL), and triglyceride levels.

**MRI Pulse Sequence Details**

The following parameters were used for the axial T1-weighted (T1W) Dixon gradient echo sequence: median echo time (TE): 2.3 ms (range, 1.2–3.9 ms), median repetition time (TR): 5.6 ms (range, 3.6–9.6 ms), median flip angle: 10° (range, 10–25°), median slice thickness: 3.0 mm (range, 2.0–5.0 mm), and median in-plane resolution: 0.25 mm² (range, 0.23–0.90 mm²). The following parameters were used for the axial T2-weighted (T2W) fast spin-echo sequence: median echo time (TE): 80.0 ms (range, 70.0–102.0 ms), median repetition time (TR): 2400.0 ms (range, 1500.0–5000.0 ms), median echo train length (ETL): 15 (range, 8–32 echoes), median slice thickness: 5.0 mm (range, 4.0–6.0 mm), and median in-plane resolution: 0.62 mm² (range, 0.46–1.50 mm²). The following parameters were used for the axial diffusion-weighted imaging (DWI) sequence: median echo time (TE): 60.0 ms (range, 45.0–90.0 ms), median repetition time (TR): 4500.0 ms (range, 3000.0–8000.0 ms), median slice thickness: 5.0 mm (range, 4.0–6.0 mm), median in-plane resolution: 1.00 mm² (range, 0.64–1.50 mm²), and diffusion b-values of 0, 100, and 800.

**Ensemble Models**

Radiomic features from the liver, spleen, and combined liver–spleen regions of interest (ROIs) were extracted from patients common to all three MRI modalities as well as their corresponding clinical features (*n* = 31 patients). Within each outer repetition (N = 50) of a stratified five-fold cross‐validation (CV), the training folds for each feature set were subjected to a nested five‐fold CV to tune the regularization parameter (C) of an L1‐penalized logistic regression. As performed in the original analysis, features with nonzero coefficients under the optimal C were retained for downstream modeling. Three classifiers (logistic regression, linear SVM, and nonlinear radial‐basis function [RBF] SVM) were trained on the scaled feature subsets. Hyperparameters for SVM models (C for linear; C and γ for RBF) were tuned via nested CV on the training folds, mirroring the procedure for feature selection. For each test fold, predicted probabilities of the given clinical outcome were obtained from each classifier for each feature set. Ensemble scores were computed by taking the arithmetic mean of the four feature set probabilities, and a threshold of 0.5 was applied to the ensemble probabilities to derive binary predictions. Performance was quantified by the area under the receiver‐operating characteristic curve (AUROC) across all outer folds and repetitions.

**Supplemental Table 1:** Baseline participant characteristics, including sub-cohorts based on MRI pulse sequence availability.

|  | **T2-Weighted**  **(*n* = 119)** | **Diffusion-Weighted**  **(*n* = 73)** | **T1-Weighted**  **(*n* = 31)** | **p** |
| --- | --- | --- | --- | --- |
| **Weight at Time of MRI (mean [SD])** | 65.75 (21.64) | 66.13 (23.02) | 65.30 (20.84) | 0.985 |
| **Height at Time of MRI (mean [SD])** | 163.18 (14.21) | 163.08 (14.07) | 162.81 (14.22) | 0.992 |
| **Creatinine (mean [SD])** | 0.75 (0.22) | 0.77 (0.23) | 0.71 (0.19) | 0.554 |
| **Albumin (mean [SD])** | 4.33 (0.53) | 4.28 (0.56) | 4.39 (0.51) | 0.643 |
| **ALT (mean [SD])** | 37.32 (23.98) | 35.31 (26.56) | 35.83 (38.00) | 0.883 |
| **AST (mean [SD])** | 27.29 (10.63) | 27.03 (11.22) | 28.55 (14.92) | 0.831 |
| **Total Bilirubin (mean [SD])** | 1.00 (0.69) | 0.92 (0.59) | 1.06 (0.69) | 0.574 |
| **ALP (mean [SD])** | 128.46 (76.41) | 111.22 (64.91) | 99.47 (50.31) | 0.085 |
| **GGT (mean [SD])** | 84.86 (88.33) | 81.79 (81.67) | 71.52 (62.85) | 0.761 |
| **INR (mean [SD])** | 1.50 (0.63) | 1.47 (0.51) | 1.32 (0.35) | 0.355 |
| **AFP (mean (SD))** | 3.49 (2.50) | 3.65 (2.64) | 3.55 (2.56) | 0.974 |
| **MELD-XI Score (mean [SD])** | 10.42 (1.82) | 10.21 (1.68) | 10.56 (1.88) | 0.700 |
| **APRI (mean [SD])** | 0.40 (0.21) | 0.42 (0.24) | 0.50 (0.34) | 0.326 |
| **Total Cholesterol (mean [SD])** | 133.89 (31.49) | 135.02 (32.31) | 140.19 (36.31) | 0.781 |
| **LDL (mean [SD])** | 72.62 (26.88) | 72.83 (26.91) | 78.56 (28.44) | 0.719 |
| **HDL (mean [SD])** | 41.57 (12.29) | 43.06 (13.33) | 40.12 (9.97) | 0.675 |

**BSA** = Body Surface Area, **ALT** = Alanine Aminotransferase, **AST** = Aspartate Aminotransferase, **ALP** = Alkaline Phosphatase, **GGT** = Gamma-Glutamyl Transferase, **INR** = International Normalized Ratio, **AFP** = Alpha-Fetoprotein, **MELD-XI** = Model for End-Stage Liver Disease Excluding INR, **APRI** = AST to Platelet Ratio Index, **LDL** = Low-Density Lipoprotein, **HDL** = High-Density Lipoprotein.
